# Contextual cueing—Eye movements in rotated and recombined displays

**DOI:** 10.3389/fcogn.2024.1403749

**Published:** 2024-05-10

**Authors:** Lei Zheng, Nico Marek, Natalia Melnik, Stefan Pollmann

**Affiliations:** ^1^Department of Psychology, Otto-von-Guericke University, Magdeburg, Germany; ^2^Center for Behavioral Brain Sciences, Otto-von-Guericke University, Magdeburg, Germany

**Keywords:** contextual cueing, oculomotor system, spatial location, attention, visual search, mental rotation, global vision, local vision

## Abstract

Contextual cueing leads to improved efficiency in visual search resulting from the extraction of spatial regularities in repeated visual stimuli. Previous research has demonstrated the independent contributions of global configuration and spatial position to contextual cueing. The present study aimed to investigate whether learned spatial configuration or individual locations would elicit fixation patterns resembling those observed in the original displays. We found that search guidance based on either local or global spatial context, by combining distractor locations from two learned displays or rotating displays, kept not only search time facilitation intact, in agreement with previous studies, but also enabled search with less fixations and more direct scan paths to the target. Fixation distribution maps of recombined or rotated displays were more similar to the original displays than random new displays. However, for rotated displays this was only true when the rotation angle was taken into account. Overall, this shows an astonishingly flexible use of the oculomotor system for search in incompletely repeated displays.

## 1 Introduction

Contextual cueing is a phenomenon characterized by the extraction of spatial regularities from repeated visual stimulus patterns, leading to enhanced efficiency of visual search. Numerous studies have consistently demonstrated the facilitation effect associated with contextual cueing, wherein participants exhibit faster search times for displays with repeated configurations (Goujon et al., 2015; Jiang and Sisk, 2019; Jiang et al., 2019; Sisk et al., 2019). Notably, previous studies have highlighted the independent contribution of spatial position and global configuration to contextual cueing (Jiang and Wagner, 2004; Zheng and Pollmann, 2019). This was achieved by the design of two types of displays: the first type of display recombined distractors from two learned configurations with the same target location, thereby preserving individual target-distractor relations but destroying the global spatial configuration of distractors. The second type of display was obtained by changing display size (Jiang and Wagner, 2004) or rotating the entire display around its center (Zheng and Pollmann, 2019), thereby preserving the spatial configuration but changing all distractor locations. Contextual cueing was observed in both types of displays, which suggests that both the spatial position of individual items and the overall arrangement of the display contribute to guiding attention and facilitating efficient search processes.

However, these studies only analyzed search times, as a summary measure of the underlying processes. Thus, although the search time benefits for recombined and rotated (or enlarged) displays demonstrated that search was more efficient in these partially repeated displays, they yielded no further information about the underlying processes. One way to learn more about these processes is to measure eye movements.

Contextual cueing not only leads to reduced search times for repeated displays but also to fewer fixations and more efficient fixation patterns (Peterson and Kramer, 2001; Tseng and Li, 2004; Brockmole and Henderson, 2006; Manginelli and Pollmann, 2009). However, in these previous studies, eye movement data were recorded in fully repeated displays. In contrast, in the present study, we explored eye movements in rotated displays—that selectively contained only the previously learned spatial configuration of items but not the learned item locations—or in recombined displays—in which all distractor locations were predictive of the target location, but the overall configuration of distractor locations was not.

There is evidence that both the individual spatial target-distractor relations and the overall spatial configuration contribute to contextual cueing. On the one hand, it was observed that repeating only distractor locations in the vicinity of the target led to search facilitation of about the same magnitude as repeating all locations of a display (Olson and Chun, 2002; Brady and Chun, 2007). On the other hand, in the same studies, effects of global context on contextual cueing were observed, e.g., the search facilitation due to contextual cueing was lost if all distractor locations in the target quadrant were preserved, but the quadrants of a display were shuffled (Brady and Chun, 2007, Exp. 4). Thus, the evidence gained from search times suggests that both the local distractor position and the global target-distractor configuration of a display are vital information sources that help predict the target location.

Here, we asked if the pattern of eye movements made during search in a recombined or rotated display would be similar to the original display that was encountered during the—incidental—learning phase of the experiment. Regarding the recombined displays, the question was, would the eye movement pattern for searching a display in which 100% of distractor locations were predictive of the target, but 50% each stemmed from two different trained displays, thus destroying the overall target-distractor configuration, still be more similar to the eye movement pattern in the original display(s), compared to an eye movement pattern recorded during viewing a new display? Regarding the rotated displays, we asked if a display that is repeated but rotated by 45° elicits a fixation pattern that is similar to the one elicited by the original display, but likewise rotated by 45°. Alternatively, it might be assumed that a rotation angle of 45° is sufficiently small that the fixation pattern from the original display need not be rotated, perhaps using larger attentional foci (indicated by fewer fixations and larger saccade amplitudes) for an efficient search.

It may be argued that higher similarity of fixation patterns for a trained display and its recombined or rotated versions, rather than a new display, is trivial, because it may simply be achieved by fixating the display items one by one. However, fixation patterns are typically not identical when a scene is repeatedly presented. Rather, the number of fixations tends to be reduced for repeated presentations (Smith et al., 2006; Damiano and Walther, 2019). We expected the same here, a fixation pattern that is similar—though perhaps rotated—to the fixation pattern during initial presentation, but reduced in the number of fixations and with a more efficient scan path, yielding a reduced search time.

## 2 Methods

### 2.1 Participants

Twenty-four young adults (13 females and 11 males) with an average age of 23.7 years participated in the experiment, having self-reported normal or corrected-to-normal vision, and provided written informed consent for taking part in this study. Data from two participants were excluded because of unexpected program crashes, resulting in a final sample size of twenty-two participants. After they completed the experiment, participants were compensated with an 8-euro payment or a 1-h study credit.

### 2.2 Equipment

Participants were individually tested in a sound-attenuated chamber, to ensure minimal external distractions. During the experiment, participants viewed a computer screen from a fixed distance of 57 cm by using a chin rest. Stimuli were presented on a screen with a resolution of 1,920 × 1,080 pixels and a refresh rate of 60 Hz.

The PsychoPy software was used to generate stimuli, control the timing of experimental events, and record participants' responses. Eye movements were recorded with an EyeLink 1000 desktop mount eye-tracking system (SR Research, Missisauga, Ontario, Canada), with 1,000 Hz temporal resolution.

### 2.3 Stimuli

**Figure 1:**
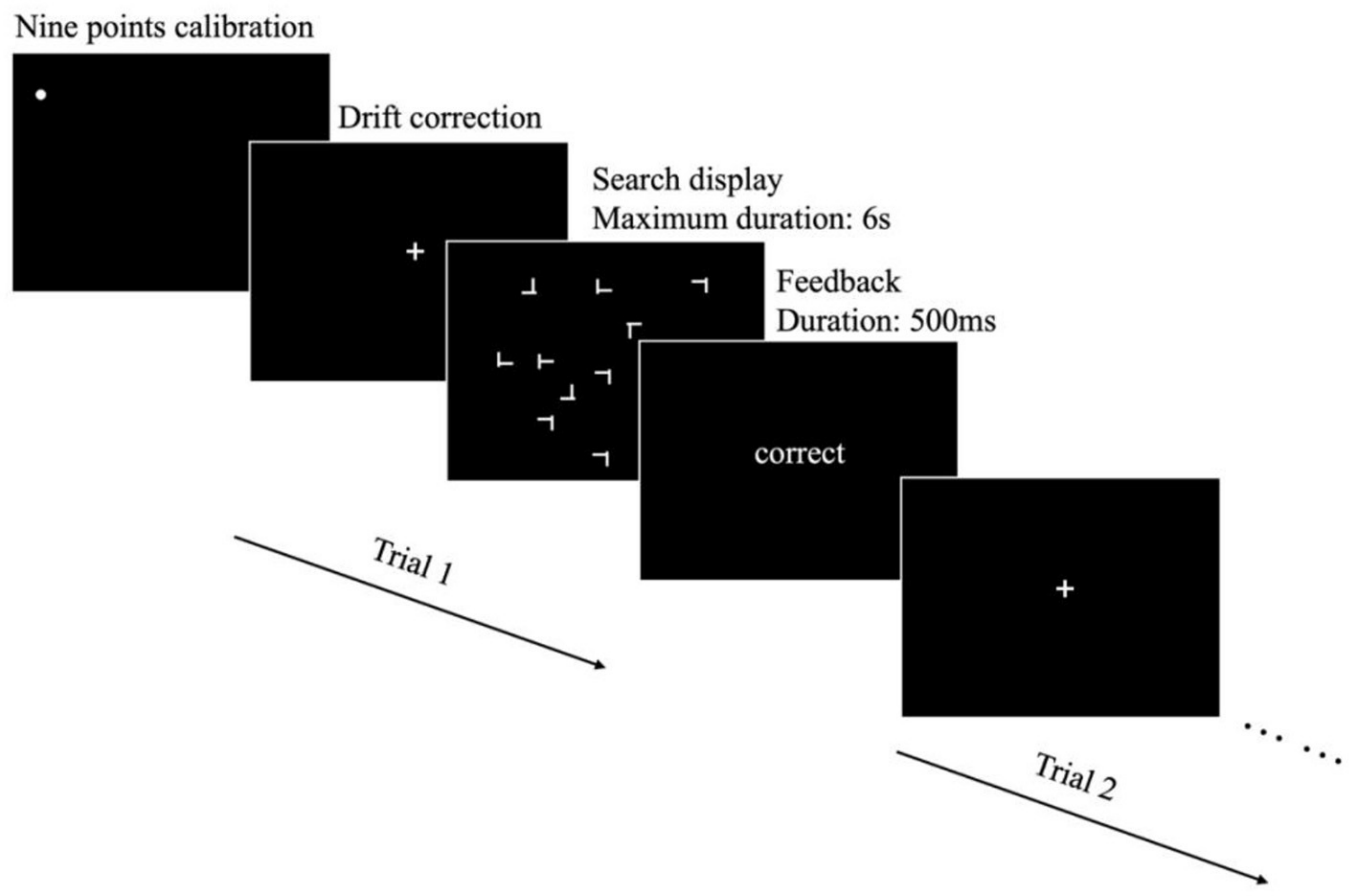
The procedure employed in the present experiment. Prior to the start of each block, a nine-point calibration was conducted to ensure the accuracy of the eye-tracking system. Throughout the block, ongoing calibration checks were performed and adjustments were made when necessary to maintain optimal tracking accuracy. Each trial began with a white fixation cross at the center of the screen, serving as an initial point of visual focus for participants. Following this, a search display was presented, where participants were required to indicate the target's rotation by pressing respective buttons. The search display remained visible until either the button was pressed or a maximum duration of 6 s had elapsed. At the end of each trial, feedback was presented for 500 ms.

The displays consisted of an arrangement of eleven white items (one T-shaped target and ten L-shaped distractors) that were presented against a black background (Figure 1). The target T was rotated by 90° to the left or right and the distractors were rotated by 0°, 90°, 180°, or 270°. Each letter's orientation was randomly determined for each trial. To increase the search difficulty, there was an offset (seven pixels) of the line junction of the distractor Ls, making the distractors more similar to the target T (Jiang and Chun, 2001).

### 2.4 Procedure

The eye tracker was calibrated with the standard nine-point calibration procedure at the start of each block. Throughout the block, recalibration was performed as required to account for any potential measurement errors or variability arising from subtle shifts in pupil position across different trials.

Each trial began with a drift correction procedure to check whether the calibration model is still accurate. Following successful drift correction, participants were presented with the search display, during which they were allowed to freely move their eyes. The task was to identify the orientation of the T target (either rotated to left or rotated to right) by pressing the corresponding left or right key on a keyboard. The search display remained visible until the participants responded, or until a maximum duration of 6 s had elapsed. Participants were given feedback after each response. This feedback provided participants with information regarding the accuracy of their responses in the preceding trial, allowing for real-time performance evaluation. The experimental procedure is illustrated in Figure 1.

### 2.5 Design

The experiment consisted of a learning phase and a transfer phase. The learning phase consisted of 20 blocks of 12 trials (240 trials in total), and the transfer phase consisted of four blocks of 48 trials (192 trials in total). To mitigate participant fatigue and allow for periodic breaks, every set of 48 trials (four blocks in the learning phase, and one block in the transfer phase) was followed by a rest period of 10 s. The whole experiment took about 50 min to complete.

#### 2.5.1 Learning phase

During the learning phase, 12 displays were repeatedly presented over 20 blocks. The positioning of the items in the experiment followed a specific arrangement of four concentric circles with radii of 2.04°, 3.99°, 6.28°, and 8.76°. Within each circle, there were eight equidistant possible item locations. To ensure that the target was not readily detectable at display onset, target locations were chosen only on the three outer circles.

The displays were generated individually for each participant, with the following procedure. Six target positions were randomly chosen on each of the three outer circles. Next, the second target position, following a 45-degree shift, was selected for each target. Overall, for six initial target locations, six rotated target locations were selected. By employing this design, this selection of target positions allowed for the systematic exchange of two target locations in the rotated condition of the transfer phase while maintaining a consistent probability distribution of the target location.

Each target position was paired with two sets of distractors in different positions. Specifically, for each target position, 20 distractor positions were randomly selected from the pool of items. These selected distractor positions were then randomly divided into two sets of ten positions. Each set of ten positions was matched with the target to form a display configuration. In this way, a total of twelve displays (six target locations × two sets of distractor locations) were generated and presented in random order in each block.

#### 2.5.2 Transfer phase

**Figure 2:**
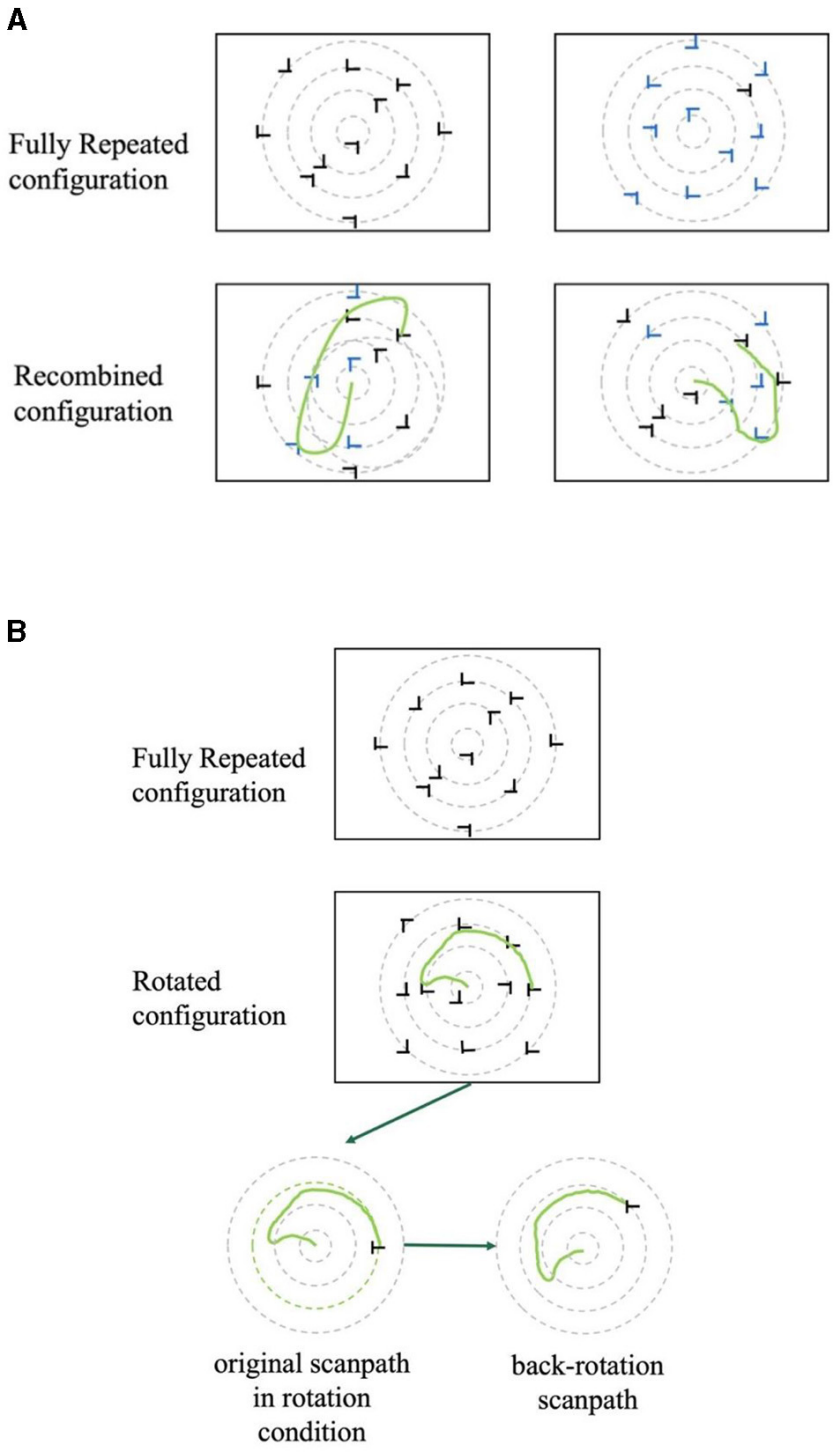
**(A)** Examples of two recombined displays with the same target location but different (symbolized by black and blue) distractors. In the actual experiment, all distractors were black. The hand-drawn green lines represent possible eye movement trajectories. **(B)** An example of a fixation pattern in a rotated display and its back-rotated version in the transfer phase.

The transfer phase consisted of four blocks. Each block consisted of 12 displays encompassing four randomly interleaved configuration types: Fully Repeated, Recombined, Rotated, and New, following the design of the prior study by Zheng and Pollmann (2019). Fully Repeated displays were identical to those presented during the learning phase. This configuration served as a baseline for evaluating the retention and transfer of learned spatial associations. In the Recombined displays, the six target locations were consistent with those of the Fully Repeated displays. However, one half of the distractors each was selected randomly from each of the two distractor sets that were initially paired with the same target location in the learning phase (Figure 2A). To create the Rotated displays, half of the Fully Repeated displays were rotated 45° clockwise, while the other half was rotated 45° counterclockwise. As the polar angle between the two target positions on each circle was 45°, the target location probabilities of the six target locations were kept constant by this manipulation (Figure 2B). Finally, New displays contained again the same six target locations as the other display types, but each target location was paired with two sets of randomly selected distractor locations. To keep learning of repeated and new displays comparable during the transfer phase, New displays were also repeated across transfer blocks.

### 2.6 Data analysis

#### 2.6.1 Accuracy and search time

Our main focus was the analysis of search times, defined as the time from display onset to the response. However, we also analyzed the rate of incorrect responses to rule out speed-accuracy trade-offs. To this end, we conducted one-way analyses of variance to analyze the proportion of trials in which participants either provided incorrect responses or failed to respond within the designated time frame (6 s). Incorrect and slow response trials were excluded from the dataset for search time analyses.

To assess whether the reduction in search time reached a plateau toward the end of the learning phase, indicating that most of the learning had occurred and little additional learning was expected in the transfer phase, a Bayesian ANOVA was conducted on blocks 16–20.

During the transfer phase, we further explored the influence of different configurations on search performance. To analyze the proportion of incorrect trials, we conducted one-way analyses of variance, with the configuration considered as fixed factor. Regarding the analysis of search time, since the main effect across blocks was of particular interest, we performed a repeated measures ANOVA, using search time as dependent variable, and block and configuration as within-subject factors. If the main or interaction effect was significant, we utilized *post-hoc* tests to investigate specific contrasts.

Equality of search times across the partially and fully repeated conditions was assessed by calculating Bayes factors for the Fully Repeated, Recombined, and Rotated configurations.

#### 2.6.2 Basic oculomotor indicators

We also conducted an examination of fundamental oculomotor metrics, specifically focusing on the number of fixations, saccade amplitude (average per participant), and scan pattern ratio [SPR, calculated by dividing the sum of saccade amplitudes by the straight-line distance from the scene center (initial fixation) to the target location; Brockmole and Henderson, 2006]. Employing a repeated-measures analysis of variance (ANOVA) with the configurations and blocks as main factor, we aimed to ascertain potential dissimilarities in these indicators within distinct configurations and across distinct time blocks. Subsequent paired-sample *t*-tests were conducted to further elucidate the significance of specific pairwise comparisons.

#### 2.6.3 Fixation density maps

**Figure 3:**
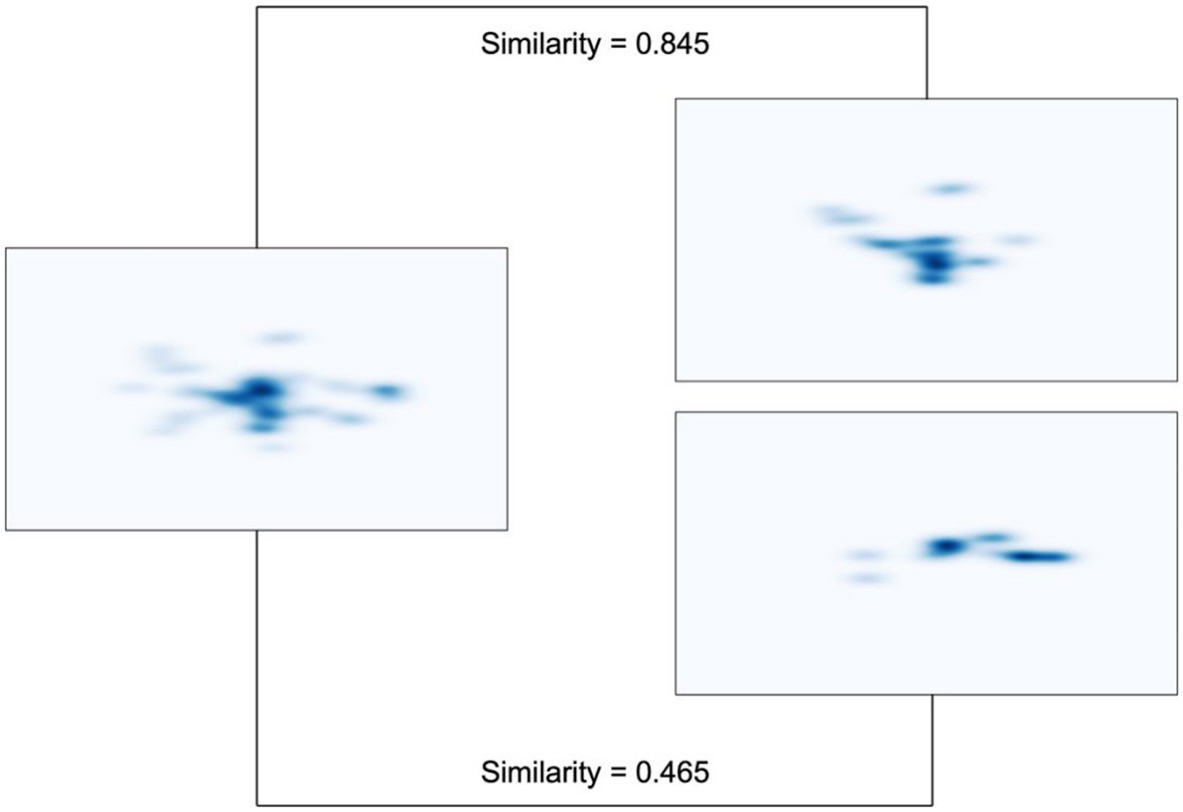
Representative examples of fixation density maps with varying degrees of similarity. The map on the left shares a higher similarity with the one on the top right (0.845), while it exhibits lower similarity with the one on the bottom right (0.465). These examples are taken from three displays belonging to one participant. The three displays share the same target location but are from different conditions: fully repeated condition **(left)**, back-rotated condition **(top right)**, and new condition **(bottom right)**.

For each trial, the spatial distribution of the eye movement data was plotted as an FDM with the same limits as the original presentation screen (1,920 × 1,080 pixel). To correct for possible inaccuracies during recording, each FDM was spatially smoothed using a Gaussian filter with sigma of 30. All FDM were saved for visual inspection, transformed into greyscale matrices using the Open Computer Vision Library and subsequently vectorized with Numpy (Harris et al., 2020). Pairwise correlation distances (1 – correlation) were calculated between all vectors. Correlation distance coefficients were then transformed into Pearson coefficients. In order to achieve an approximately normal distribution, Fisher's *z*-transformation was applied. Figure 3 illustrates representative examples of relatively similar and dissimilar fixation density maps.

The similarity in FDM was assessed using paired *t*-tests, comparing fully repeated with the other two partially repeated conditions. In addition, we tested the hypothesis that participants were able to mentally rotate a learned display configuration to adapt it to a rotated display. In this case, the fixation pattern may also be rotated to adapt to the display rotation. Consequently, a back-rotated version of the fixation pattern should fit the fixation pattern of the same displays fully repeated (i.e., unrotated) version better than the fixation pattern before back-rotation. The back-rotated condition was created by rotating the fixations from the rotated displays in the direction opposite to display rotation by 45 degrees, as depicted in Figure 2B. Overall, this FDM analysis involved comparisons between fully repeated and recombined displays, rotated and back-rotated displays, as well as fully repeated and back-rotated displays.

## 3 Results

### 3.1 Learning phase

**Figure 4:**
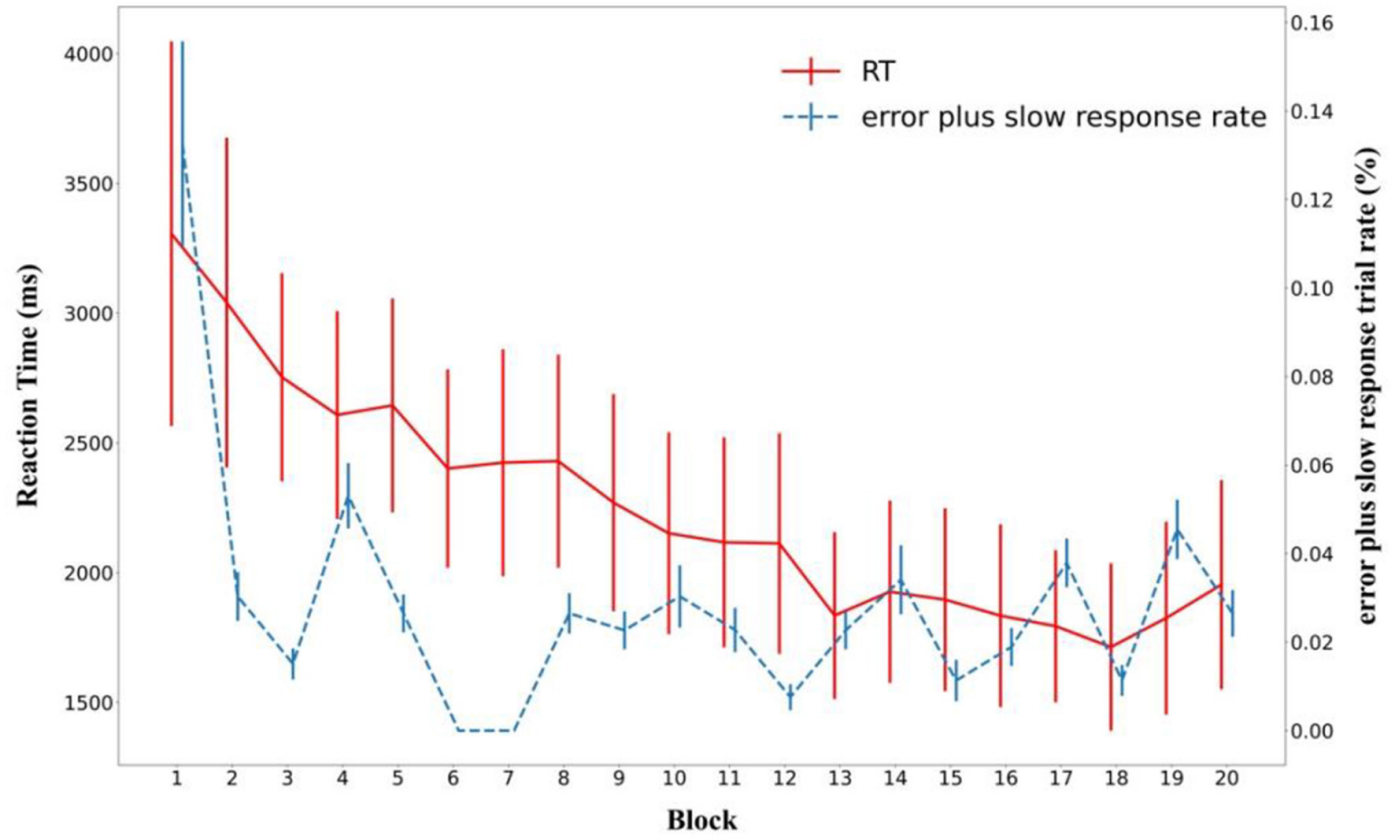
Mean search time and rate of incorrect plus timeout (slow) responses during the learning phase across blocks. Error bars represent standard errors of the mean.

The participants exhibited high performance, with error (including slow response) trials rate of only 2.88% throughout the learning phase. The rate of error including slow response trials decreased as the experiment progressed (Figure 4), confirmed by a significant block main effect in a one-way analysis of variance [*F*_(19, 420)_ = 2.798, *p* < 0.001, $\eta_{p}^{2}$ = 0.112].

Importantly, search times exhibited a significant decrease over the course of the experiment as well (Figure 4). A one-way ANOVA showed a main effect of Block [*F*_(19, 420)_ = 6.052, *p* < 0.001, $\eta_{p}^{2}$ = 0.215].

However, Figure 4 suggests that search times plateaued toward the end of the learning phase. This was strongly supported by a Bayesian ANOVA over the last five blocks (BF_01_ = 18.7).

### 3.2 Transfer phase

#### 3.2.1 Error (including slow response) trials rate

In the transfer phase, the error (including slow response) trials trial rate was measured across four conditions, namely Fully Repeated, Recombined, Rotated, and New. The incorrect response rates in each condition were low overall (3.21% for Fully Repeated, 3.23% for Recombined, 3.79% for Rotated, and 5.11% for New) and a one-way ANOVA did not yield a significant main effect of condition [*F*_(3, 84)_ = 1.056, *p* = 0.372].

#### 3.2.2 Search times

The repeated measures ANOVA with the configuration and block as factors revealed significant main effects of Configuration [*F*_(3, 63)_ = 7.875, *p* < 0.001, $n_{p}^{2}$ = 0.273] and Block [*F*_(3, 63)_ = 4.925, *p* = 0.004, $n_{p}^{2}$ = 0.190] with search times decreasing over blocks. The interaction between Configuration and Block was not significant [*F*_(9, 189)_ = 0.292, *p* = 0.976, $n_{p}^{2}$ = 0.014].

**Figure 5:**
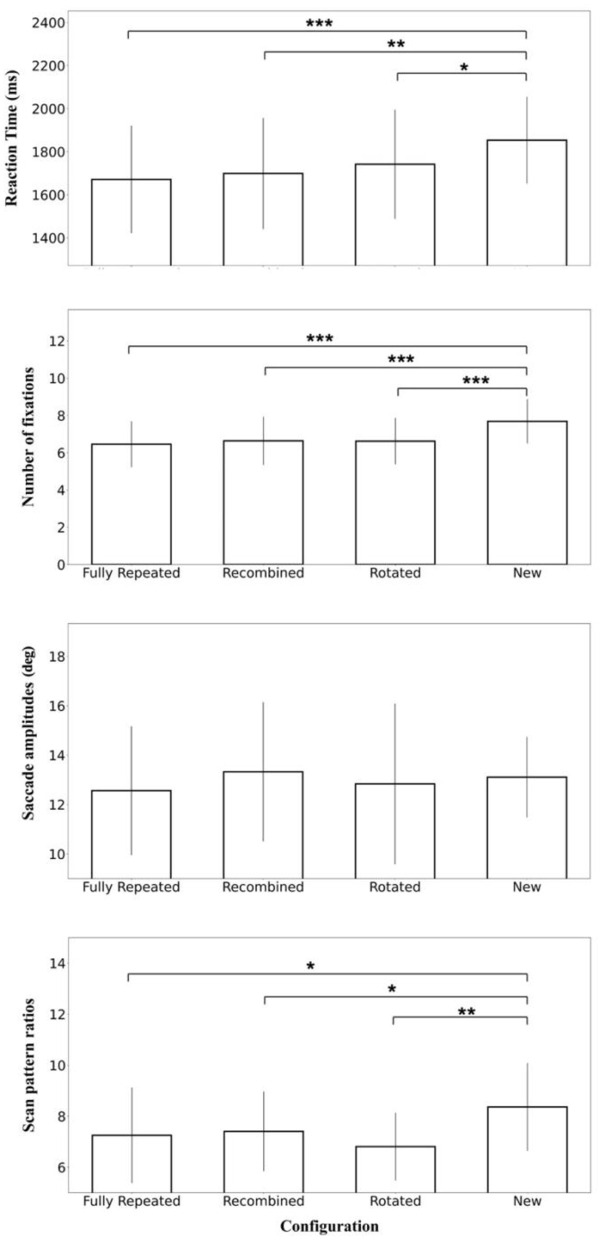
Mean search time, the number of fixations, saccade amplitudes, and scan pattern ratios in the four configurations (fully repeated, recombined, rotated, and new) of the transfer phase. Error bars represent standard errors of the mean. Significance levels are denoted by asterisks, with *p < 0.05; **p < 0.01, ***p < 0.001.

The *post-hoc* analysis showed that the search times in the New configuration were significantly higher than in the Fully Repeated configuration [*t*_(21)_ = 4.509, *p* < 0.001, *d* = 0.376], the Recombined configuration [*t*_(21)_ = 3.827, *p* = 0.002, *d* = 0.319], and the Rotated configuration [*t*_(21)_ = 2.764, *p* = 0.045, *d* = 0.231], indicative of contextual cueing in the three (partially) repeated configurations. No other comparisons yielded significant results (Figure 5).

To test for equality of search times between the repeated conditions, Bayes factors were calculated for the Fully Repeated, Recombined, and Rotated configurations. The Bayes factor (BF_01_) of 1.996 between Recombined and Rotated and 1.081 between Fully Repeated and Rotated yielded anecdotal evidence for equality. Additionally, the Bayes factor of 3.574 between Fully Repeated and Recombined yielded substantial evidence for equality (Wetzels et al., 2011).

#### 3.2.3 Basic oculomotor indicators

##### 3.2.3.1 Number of fixations

The repeated measures ANOVA with the configuration and block as main effect on the number of fixations revealed significant main effects of Configuration [*F*_(3, 63)_ = 17.903, *p* < 0.001, $n_{p}^{2}$ = 0.460] and Block [*F*_(3, 63)_ = 3.284, *p* = 0.026, $n_{p}^{2}$ = 0.135] with fewer fixations over blocks. The interaction between Configuration and Block was not significant [*F*_(9, 189)_ = 1.229, *p* = 0.279, $n_{p}^{2}$ = 0.055].

The *post-hoc* analysis showed that the number of fixations in the new configuration was significantly higher than in the fully repeated configuration [*t*_(21)_ = 5.516, *p* < 0.001, *d* = 1.176], the recombined configuration [*t*_(21)_ = 5.534, *p* < 0.001, *d* = 1.180], and the rotated configuration [*t*_(21)_ = 6.060, *p* < 0.001, *d* = 1.292]. No other comparisons yielded significant results (Figure 5).

##### 3.2.3.2 Saccade amplitudes

The repeated measures ANOVA on saccade amplitude did not indicate significant main effects of configuration [*F*_(3, 63)_ = 0.513, *p* = 0.675, $n_{p}^{2}$ = 0.024] or block [*F*_(3, 63)_ = 1.543, *p* = 0.212, $n_{p}^{2}$ = 0.068]. The interaction between configuration and block [*F*_(9, 189)_ = 0.340, *p* = 0.960, $n_{p}^{2}$ = 0.010] was also not significant (Figure 5).

##### 3.2.3.3 Scan pattern ratios

The repeated measures ANOVA on scan pattern ratios revealed a significant main effect of configuration [*F*_(3, 63)_ = 4.397, *p* = 0.007, $n_{p}^{2}$ = 0.173]. The main effect of block [*F*_(3, 63)_ = 2.160, *p* = 0.102, $n_{p}^{2}$ = 0.093] and the interaction between Configuration and Block were not significant [*F*_(9, 189)_ = 1.606, *p* = 0.116, $n_{p}^{2}$ = 0.071].

The *post-hoc* analysis showed that the scan pattern ratios in the new configuration were significantly higher than in the fully repeated configuration [*t*_(21)_ = 1.835, *p* = 0.040, *d* = 0.391], the recombined configuration [*t*_(21)_ = 2.199, *p* = 0.020, *d* = 0.469], and the rotated configuration [*t*_(21)_ = 3.025, *p* = 0.003, *d* = 0.645]. No other comparisons yielded significant results (Figure 5).

#### 3.2.4 Spatial fixation density maps

**Figure 6:**
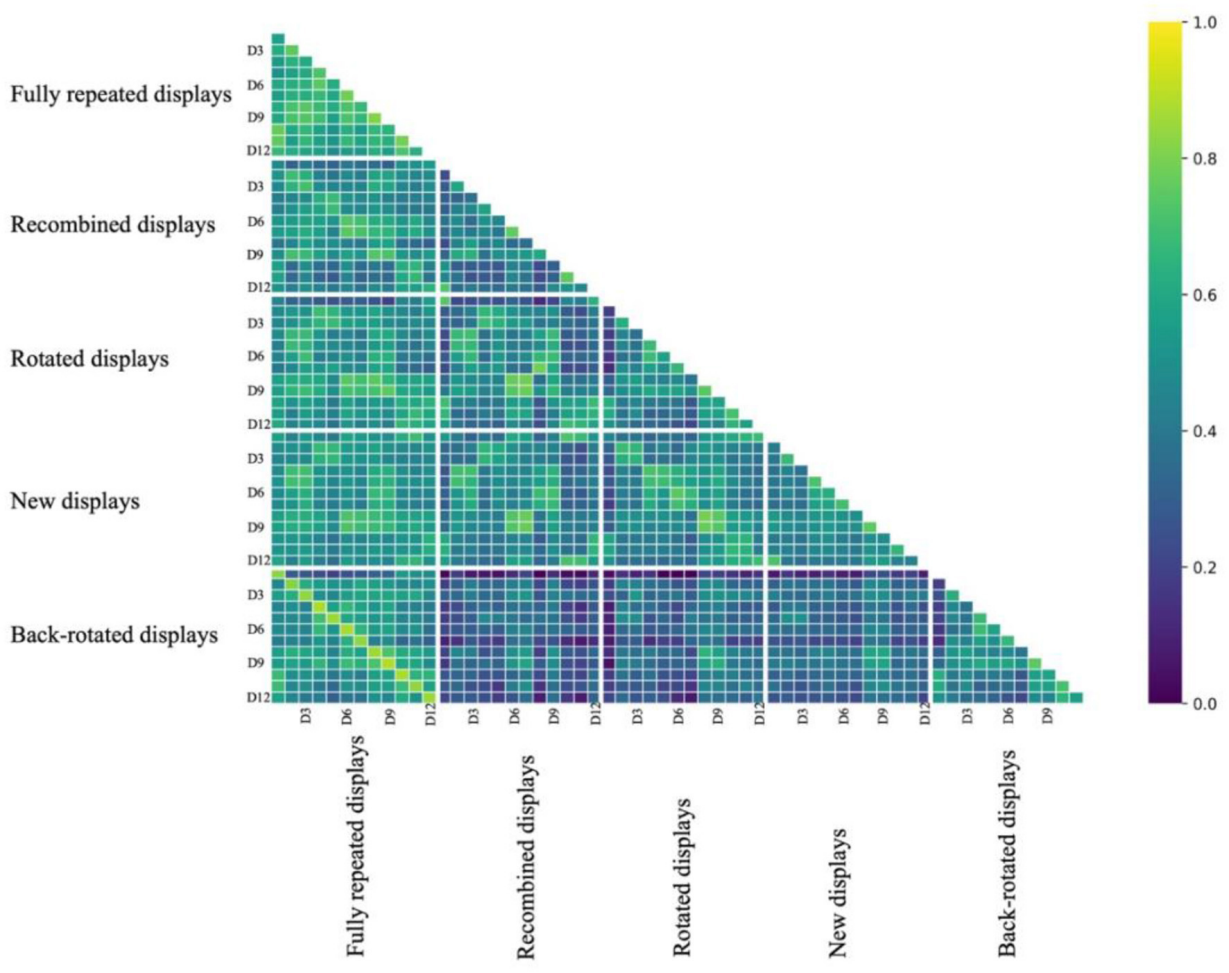
The figure depicts the similarity matrix of 12 displays across five conditions, with distinctive white lines marking the boundaries between different conditions. In the horizontal and vertical axes, “D” represents “display”. Each correlation coefficient in the matrix was converted back from the distance correlation (1–r) and then transformed using the Fisher *Z*-transformation.

We compared FDMs to analyze the similarity of the spatial fixation distribution between conditions. Figure 6 shows the similarity matrix of 12 displays and five conditions.

What can be seen in the matrix is that similarity was high in the lines parallel to the main diagonal, indicating that displays sharing the same target location and parts of the distractor locations (i.e., fully repeated and recombined displays) had more similar FDMs than displays not sharing these locations. This was most obvious for the similarity of back-rotated FDMs and fully repeated FDMs, where an individual display and its back-rotated version shared the target and all distractor locations.

In particular, we expected recombined displays to elicit FDMs similar to those of the fully repeated displays they received one half of the distractors from. Consequently, we anticipated that the FDMs of the fully repeated displays were more similar to the FDMs of the respective recombined displays than to those of the new displays sharing the same distractor location. This was confirmed by a *t*-test comparison, indicating the similarity between repeated and recombined displays was higher than that between the fully repeated and new displays [*t*_(21)_ = 6.276, *p* < 0.001, *d* = 1.338].

We further expected that a rotated display would prompt FDM similar to those of the original display, albeit rotated by the angle of display rotation. Thus, we expected the back-rotated FDMs to be more similar to the FDM for the fully repeated presentation of the same display than the rotated FDM. A *t*-test comparison revealed that the similarity between repeated and back-rotated displays was higher than that between the repeated and rotated displays [*t*_(21)_ = 15.490, *p* < 0.001, *d* = 3.302].

In addition, in order to confirm that the similarity of back-rotated FDMs of rotated displays and the FDMs of fully repeated displays are not just driven by shared (though rotated) target location, they should be more similar to fully repeated displays than new displays with the same target location. Again, this was confirmed through a *t*-test, revealing that the similarity between repeated and back-rotated displays was higher than that between the repeated and new displays [*t*_(21)_ = 10.200, *p* < 0.001, *d* = 2.175].

**Figure 7:**
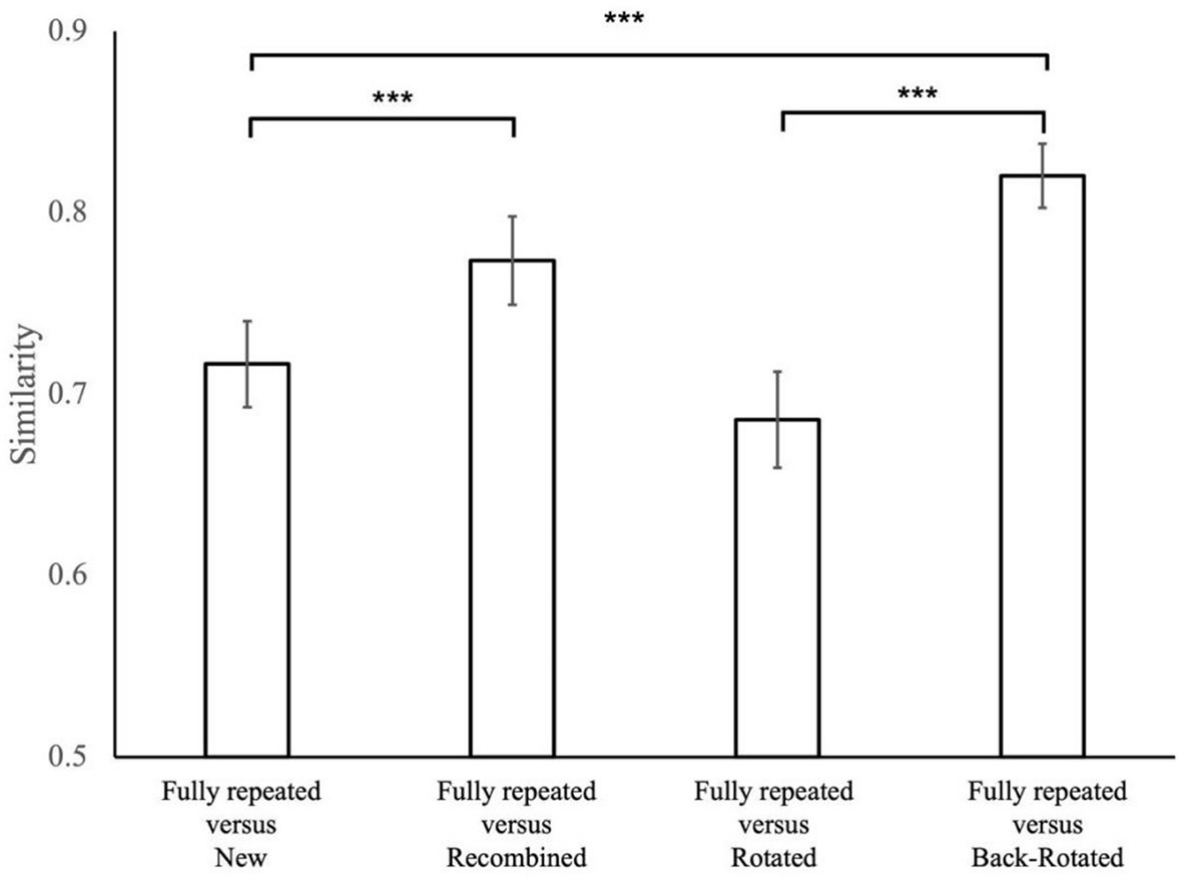
The similarity values for condition comparisons in the present study. Error bars represent standard errors of the mean. The significance level in result of *t*-tests is denoted by asterisks, with ***p < 0.001.

The results of paired *t*-tests are visually presented in Figure 7, with specific values detailed earlier in the text.

Looking at the similarity matrix (Figure 6), it appears that fixation patterns between different fully repeated displays are more similar than between different new displays. This is puzzling because using learnt distractor configurations for search guidance might be expected to lead to more distinct fixation patterns for repeated displays. Recently, however, it has been proposed that the search time benefits of repeated displays in the contextual cueing paradigm come about not (only) due to search guidance enabled by the learnt configurations of individual displays, but by a common search path that is optimized for all repeatedly presented displays (Seitz et al., 2023). This common scanpath hypothesis predicts that search paths—and consequently fixation patterns—of different repeated displays should be more similar than between different new displays. We indeed found that the similarity of fixation patterns was significantly higher between different fully repeated displays than between different new displays [*t*_one − sided_ (21) = 14.667, *p* < 0.001, *d* = 3.127]. Thus, the fixation patterns might support the “common scanpath” hypothesis. However, the similarity of fixation patterns between rotated displays were not significantly higher than between new displays, {rotated: [*t*_two − sided_ (21) = −1.843, *p* = 0.079]}, and, the similarity between recombined displays was significantly lower than between new displays [*t*_two − sided_ (21) = −4.081, *p* < 0.001, *d* = −0.870].

## 4 Discussion

Search in repeated displays can not only lead to reduced search times, but also to more efficient scan paths during visual search (Peterson and Kramer, 2001; Tseng and Li, 2004; Brockmole and Henderson, 2006; Manginelli and Pollmann, 2009). Here, we investigated if that is also the case if only partial spatial constellations are repeated in recombined displays or the display is rotated. We found that recombined displays, containing repeated target and distractor positions, but lacking a repeated global configuration, were searched with less fixations and an improved scan pattern ratio than new displays. Furthermore, spatial fixation heat maps in these displays were more similar to those of fully repeated displays than to novel displays.

Likewise, search in rotated displays, in which the global configuration was preserved, but local target and distractor positions were changed, were also searched with less fixations and an improved scan pattern ratio. Spatial fixation heatmaps showed that this was achieved by rotating the fixation pattern along with the display rotation.

Visual search benefits from repeated spatial patterns, even if they are incomplete. For instance, previous work had already shown that repetition of three items out of a previously learned display were sufficient to elicit a search advantage, as an indicator of contextual cueing (Song and Jiang, 2005; Bergmann and Schubö, 2021). Likewise, displays that conserved only spatial relations between individual distractor and target locations by recombining distractor locations from two learned displays with the same target location on the one hand or only global target-distractor configurations on the other hand (by magnification or rotation) elicited contextual cueing (Jiang and Wagner, 2004; Zheng and Pollmann, 2019). The reduced search times found in the latter studies, indicating the use of learned spatial relations for efficient search guidance, were replicated in the present study. In addition, analysis of eye movements showed that the increased search efficiency in both recombined and rotated displays was achieved by making fewer fixations that led more straightforward to the target—indicated by improved scan pattern ratio. This extends previous reports of more efficient eye movement patterns in fully repeated displays (Peterson and Kramer, 2001; Tseng and Li, 2004; Brockmole and Henderson, 2006; Manginelli and Pollmann, 2009) to displays that repeat only local or global spatial context. Note that all four conditions—fully repeated, recombined, rotated, and new—shared the same target locations, so that differences in search efficiency between these conditions cannot be due to target location probability cueing (Geng and Behrmann, 2005; Jiang et al., 2013; Golan and Lamy, 2023).

Fixation heatmaps of recombined displays were more similar to those of fully repeated displays than those of new displays. It might be argued that this is trivial, because recombined and fully repeated displays share half of the distractor locations, whereas fully repeated and new displays share only a few randomly drawn distractor locations, if any. Although fixation need not fall on items, but rather on points that allow to judge item relations important for the task at hand, the partially joint structure may well explain the similarity of fixation heatmaps for fully repeated and recombined displays. However, in combination with the reduced number of fixations and the improved scan pattern ratio, the similarity of fixation heatmaps for recombined and fully repeated displays shows that during search of recombined displays, participants do not just follow the items on the screen, but extract useful information from the previously encountered spatial target-distractor relations to guide search efficiently. Importantly, however, the spatial relation of individual distractors and the target appears to be only useful for contextual cueing if no random distractors lie in between repeated target-distractor pairs (Olson and Chun, 2002).

Concerning the rotated displays, analysis of fixation heatmaps yielded important information that would not have been available by investigating fixation counts and scan pattern ratios alone. We found that the spatial fixation heatmaps of rotated displays were comparably dissimilar to the fixation heatmaps of the identical, but unrotated (fully repeated) displays as new displays. However, when we back-rotated the heatmaps of the rotated displays, they became significantly more similar to the non-rotated displays than the new displays, indicating that our participants appeared to rotate their learned scan patterns to efficiently search in rotated displays.

Unexpectedly, we found that fixation patterns were more similar between different fully repeated displays than between different new displays. If fixations patterns followed incidentally learnt display configurations, one might expect the opposite, namely distinctive fixation patterns for individual repeated displays. Recently, an alternative explanation has been proposed to account for the search time advantage of repeated displays. Seitz et al. (2023) proposed that a common search path is learned that is optimal for all repeated displays, rather than different search paths for individual displays. Our finding of increased fixation pattern similarity between fully repeated displays may support this assumption. However, fixation pattern similarity was not increased between recombined displays or rotated displays. Thus, the search time reductions observed for these displays could not be explained by the concept of a common search path. It seems likely that if learning a common search path contributes to the contextual cueing effect, it may not be the only contributing mechanism. As this was a *post-hoc* finding, it should be regarded with caution. However, we find it intriguing enough to warrant further research.

Neither for rotated nor recombined displays did we observe altered saccade amplitudes. Increased saccade amplitudes, together with a reduced number of fixations, might have been a sign of a search with an enlarged focus of attention (Geringswald and Pollmann, 2015). This might have been a way to capture the global configuration in altered displays. In contrast, a reduced focus of attention might have been a possible reaction to the conserved spatial relations of individual target-distractor locations in the absence of a preserved global configuration. However, as noted, we observed no indication of such adjustments. Note, that fully repeated, recombined and rotated displays were presented in random sequence, so that a deliberate advance selection of an attentional focus size to focus on more global or local spatial contexts would have been impossible.

We did not see significant differences in the search times to fully repeated, recombined or rotated displays. However, we do not know if a 50% repetition of distractor locations in the recombined displays and a 45-degree rotation of the configuration in the rotated displays equate to the same degree of disruption of the global configuration and individual locations. A more nuanced exploration of these aspects could contribute to a comprehensive understanding of the observed phenomena and guide future research in this domain.

In summary, we found that eliminating either local or global spatial context from repeated search displays kept not only search time facilitation intact, but was also accompanied by less fixations and a more direct scan path to the target. Fixation distribution maps of recombined or rotated displays were more similar to the original displays than random new displays. However, for rotated displays this was only true when the rotation angle was taken into account. Overall, this shows an astonishingly flexible use of the oculomotor system for search in incompletely repeated displays.

## Data availability statement

The data are available at https://osf.io/k6hxb/.

## Ethics statement

The studies involving humans were approved by Ethics Board of the Medical Faculty of the Otto-von-Guericke University, Magdeburg. The studies were conducted in accordance with the local legislation and institutional requirements. The participants provided their written informed consent to participate in this study.

## Author contributions

LZ: Formal analysis, Investigation, Writing – original draft. NMa: Formal analysis, Writing – review & editing. NMe: Writing – review & editing. SP: Supervision, Writing – review & editing.
